# miR-150 exerts antileukemia activity *in vitro* and *in vivo* through regulating genes in multiple pathways

**DOI:** 10.1038/cddis.2016.256

**Published:** 2016-09-22

**Authors:** Zhi Hong Fang, Si Li Wang, Jin Tao Zhao, Zhi Juan Lin, Lin Yan Chen, Rui Su, Si Ting Xie, Bing Z Carter, Bing Xu

**Affiliations:** 1 Department of Hematology, The First Affiliated Hospital of Xiamen University, Xiamen 361003, China; 2 Section of Molecular Hematology and Therapy, Department of Leukemia, The University of Texas M. D. Anderson Cancer Center, Houston, TX, USA

## Abstract

MicroRNAs, a class of small noncoding RNAs, have been implicated to regulate gene expression in virtually all important biological processes. Although accumulating evidence demonstrates that miR-150, an important regulator in hematopoiesis, is deregulated in various types of hematopoietic malignancies, the precise mechanisms of miR-150 action are largely unknown. In this study, we found that miR-150 is downregulated in samples from patients with acute lymphoblastic leukemia, acute myeloid leukemia, and chronic myeloid leukemia, and normalized after patients achieved complete remission. Restoration of miR-150 markedly inhibited growth and induced apoptosis of leukemia cells, and reduced tumorigenicity in a xenograft leukemia murine model. Microarray analysis identified multiple novel targets of miR-150, which were validated by quantitative real-time PCR and luciferase reporter assay. Gene ontology and pathway analysis illustrated potential roles of these targets in small-molecule metabolism, transcriptional regulation, RNA metabolism, proteoglycan synthesis in cancer, mTOR signaling pathway, or Wnt signaling pathway. Interestingly, knockdown one of four miR-150 targets (*EIF4B*, *FOXO4B*, *PRKCA*, and *TET3*) showed an antileukemia activity similar to that of miR-150 restoration. Collectively, our study demonstrates that miR-150 functions as a tumor suppressor through multiple mechanisms in human leukemia and provides a rationale for utilizing miR-150 as a novel therapeutic agent for leukemia treatment.

Hematopoiesis is a highly regulated process in which the self-renewal of hematopoietic stem cells (HSCs) and their differentiation into specific cell lineages are controlled by a series of molecular events. Either deregulated self-renewal or lose differentiation capacity of HSC can lead to the development of leukemia. Despite extensive studies, the pathophysiology of leukemia is not fully understood and the long-term prognosis remains poor in many types of leukemia. Over the past decade, microRNAs (miRNAs), a novel class of small noncoding RNA and epigenetic regulators, have been identified. Many of them are shown to participate in the regulatory network of hematopoiesis and their dysregulation is associated with hematologic malignancies.^1, 2^ Some miRNAs have been identified as oncogenes during leukemogenesis, whereas others are known as tumor suppressors.^1, 2^ They may hence be used as therapeutic tools.^3^

miR-150 has been reported to be differentially expressed in different stage of hematopoiesis and frequently deregulated in various types of hematopoietic malignancies.^4^ Numerous studies revealed dynamic changes of miR-150 expression in the development of lymphoid lineage, where miR-150 is first downregulated in B- and T-progenitor cells, later upregulated as they differentiate toward mature cells,^5, 6^ and then downregulated again during T-cell development from naive T cells to Th1/Th2 effector cells.^7^ In addition, miR-150 is thought to control the development of myeloid lineage by fine-tuning the transcription factor c-Myb and its downstream products promoting differentiation of HSC toward megakaryocytes rather than erythrocytes.^8, 9^ Furthermore, thrombopoietin, the primary humoral regulator of thrombopoiesis, downregulates c-Myb expression via increasing the level of miR-150 in megakaryocyte/erythrocyte precursors,^10^ whereas sustained depression of miR-150 favoring terminal erythropoiesis.^11^ Given the important role of miR-150 in normal hematopoiesis, it is not surprising that aberrant expression of miR-150 is frequently observed in various types of hematopoietic malignancies.^4^ Downregulation of miR-150 has been frequently observed in chronic myeloid leukemia (CML), acute myeloid leukemia (AML), and lymphoma, whereas its upregulation has been reported in myelodysplastic syndrome and chronic lymphocytic leukemia.^4^ Recently, an essential role for miR-150 was identified in the pathogenesis of MLL-rearranged AML. The repression of miR-150 maturation by MLL-fusion genes accelerated leukemogenesis in an MLL/AF9 murine model and miR-150 expression in this model inhibited leukemia cell growth through targeting *MYB* and *FLT3.*^12^ The critical tumor suppressor role of miR-150 in MLL-rearranged AML was further confirmed by a subsequent, independent study, in which *CBL* and *EGR2* were also identified as bona fide targets of miR-150.^13^ These data suggest that miR-150 acts as a tumor suppressor in certain leukemias and might have therapeutic potentials in treating leukemia.

An estimated 20–30% of human genes could be miRNA targets and each miRNA is predicted to regulate hundreds or more genes.^14, 15^ However, the current knowledge of miRNAs in the pathogenesis of leukemia is limited. Different algorithms have been developed to identify miRNA targets (for reviews see Leitão *et al.*^16^), but only few of these predictions have been experimentally validated. To better understand the biological function and mechanisms of action of miR-150 in leukemia, we investigated the role of miR-150, analyzed the effects of miR-150 on transcriptome empowered by microarray and bioinformatics, and functionally validated a number of miR-150 target genes in leukemia cells.

## Results

### miR-150 is deregulated in human leukemia

**Figure 1:**
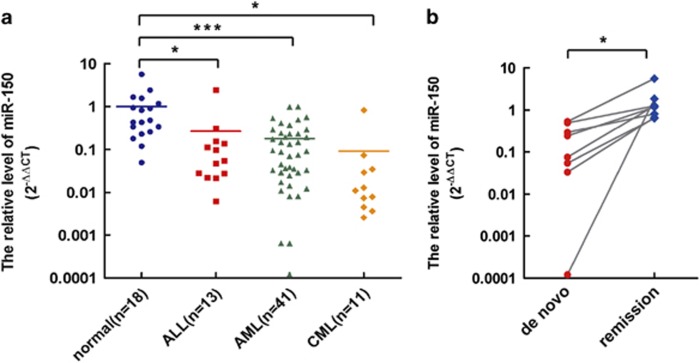
Downregulation of miR-150 in human leukemia. (**a**) miR-150 expression in the bone marrow of leukemia patients and healthy controls (*P<0.05; ***P<0.001). (**b**) Restoration of miR-150 expression in AML patients after achieving complete remission in induction chemotherapy (*n*=8). The results are expressed as miRNA expression detected by real-time qRT-PCR assay following normalization to U6 and −ΔCt calculations

To confirm the deregulation of miR-150 in leukemia, we analyzed miR-150-expressing profile in samples from various *de novo* human leukemia at the diagnosis including ALL, AML, and CML, and healthy controls using quantitative RT-PCR (qRT-PCR). The expression levels of miR-150 were markedly decreased in ALL, AML, and CML, compared with that of healthy controls (Figure 1a). We also assessed its expression in leukemia patients with bone marrow remission and found that the miR-150 level was similar to that of normal controls (Figures 1b; 1.65±1.63 *versus* 1.00±1.32, *P*=0.29). These results indicated that dysregulation of miR-150 is ubiquitous in ALL, AML, and CML.

### miR-150 suppresses cell growth and induces apoptosis in leukemia cell lines and primary AML samples

**Figure 2:**
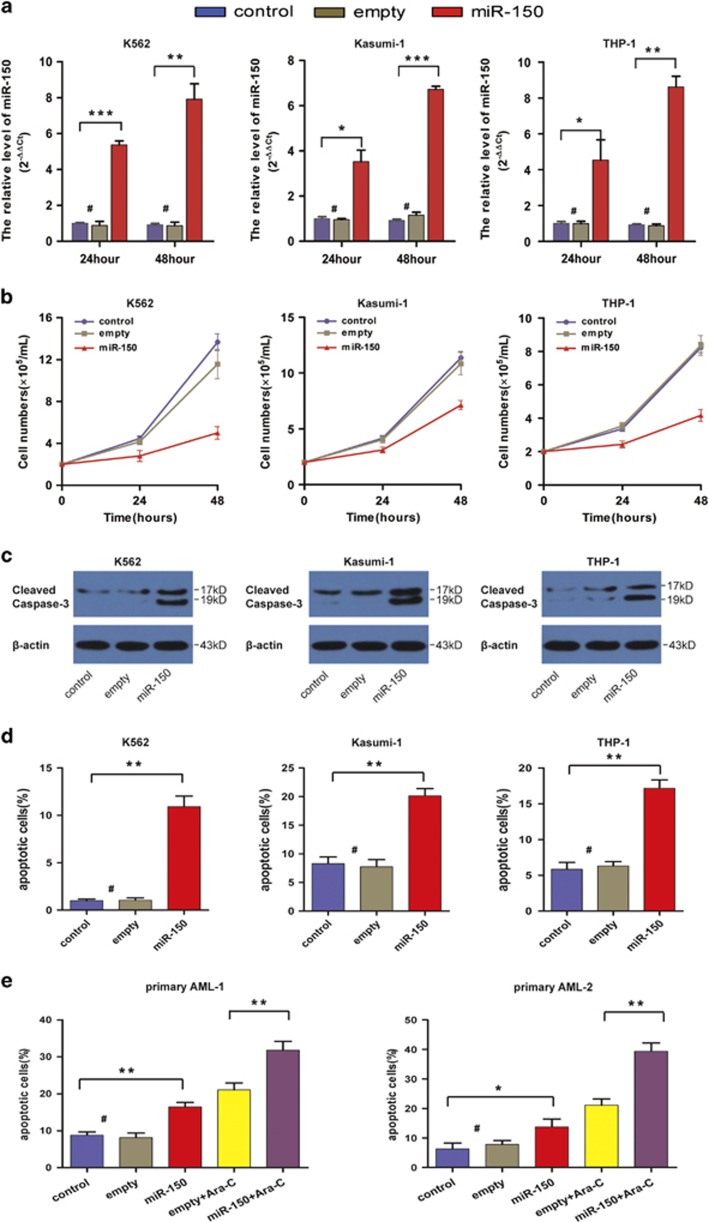
miR-150 suppresses cell proliferation and induces apoptosis in leukemia cell lines and primary AML samples. (**a**) miR-150 expression in leukemia cell lines and in the cell lines infected with miR-150. (**b**) Cell growth curves of K562, Kasumi-1, and THP-1 cells infected *in vitro* with miR-150, and their respective controls. (**c**) Caspase-3 activation in leukemia cell lines 48 h after miR-150 or empty vector transfection determined by immunoblotting. (**d**) Annexin V/7-AAD assays in leukemia cell lines 48 h after miR-150 or empty vector transfection. The results are presented as percentage of apoptotic cells. (**e**) Apoptosis analysis in two primary AML samples after 48 h of transfection with miR-150 or empty vector in the presence or absence of 5 *μ*M of Ara-C. Data are mean±S.D. of triplicates assays. *P<0.05; **P<0.01; ***P<0.001; #, no significance

To explore the biological effects of miR-150 in leukemia, we overexpressed miR-150 in three leukemia cell lines (K562, Kasumi-1, and THP-1) by lentiviral infection (Figure 2a) and examined cell growth and viability. As shown in Figure 2b, the overexpression of miR-150 inhibited the cell growth of all three leukemia cell lines. These results were validated using the CCK8 test (Supplementary Figure S1). Moreover, miR-150 overexpression promoted caspase-3 activation and increased spontaneous apoptosis in these cells (Figures 2c and d). Next, we determined whether miR-150 overexpression sensitizes the antileukemia effect of cytarabine (Ara-C), a drug commonly used in the treatment of leukemia. Three cell lines were infected with lentivirus-containing miR-150 construct and cultured with Ara-C for 24 and 48 h. Cell viability, as determined by CCK8 test, decreased in a time-dependent manner after miR-150 or/and Ara-C treatment, and an enhanced antileukemia effect was observed in the miR-150 plus Ara-C group (Supplementary Figure S2). Finally, we infected primary AML samples (*n*=2) with miR-150 lentivirus or empty lentivirus vector and treated them with Ara-C. Both miR-150 and Ara-C induced apoptosis in primary leukemia cells. Similarly, miR-150 synergized with Ara-C in apoptosis induction (Figure 2e).

### miR-150 inhibits leukemic growth in a xenograft murine model

**Figure 3:**
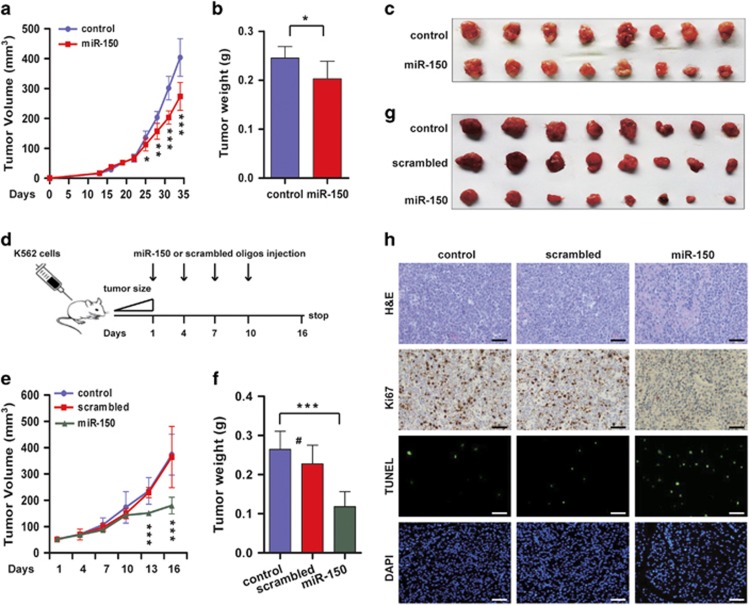
miR-150 inhibits leukemia growth in a xenograft murine model. (**a**) Tumor volumes at the indicated days during the experiment for miR-150 and control groups (*n*=8 per group). (**b**) Tumor weights of control and miR-150-overexpressing mice groups. *P<0.05. (**c**) Tumor sizes at the end of the experiment (day 34). (**d**) The experimental design of the xenografted mice treated with miR-150 or scrambled oligonucleotides. (**e**) Tumor volumes at the indicated days with miR-150 or scrambled oligonucleotide injection and the non-treatment control group (*n*=8 per group). (**f**) Tumor weights of each group at the end of experiment. ***P<0.001; #, no significance. (**g**) Tumor sizes after miR-150 or scrambled oligonucleotides treatment. (**h**) Tumor tissue histology of sections obtained from tumor-bearing mice with different treatments. Tumor morphology was analyzed by hematoxylin and eosin, cell proliferation by Ki67 staining, and apoptosis by TUNEL assay. Scale bar, 20 *μ*m. The results of one mouse from each group were shown

To further validate the tumor-suppressing role of miR-150 in leukemia, we tested whether miR-150 could reduce tumorigenicity in a xenograft model. A total of 5 × 10^6^ viable K562 cells transiently overexpressing miR-150 and its counterpart control cells were inoculated subcutaneously in the flank of immunocompromised nude mice (eight per group). The tumor growth was significantly slower after 25 days in the group overexpressing miR-150 compared with the control group (Figure 3a). At day 34, the average tumor weights for the miR-150 and the control groups were 0.20 g±0.04 and 0.24 g±0.02, respectively (*P*=0.01; Figures 3b and c). This moderate effect might be because miR-150 expression levels decreased gradually as tumor growing (Supplementary Figure S3a). Next, we inoculated parental K562 cells into mice. When the tumor volume reached ~50 mm^3^ (day 19), we injected the synthetic miR-150 or scramble oligonucleotides directly into the tumors at days 1, 4, 7, and 10 for a total of four doses (Figure 3d). We were able to detect a high level of miR-150 in the tumors (Supplementary Figure S3b). As shown in Figure 3e, at 13 days after the first injection, tumors injected with miR-150 were significantly smaller than the ones injected with scrambled oligonucleotide or controls injected with the equivalent PBS (*P*<0.001). At day 16, the average tumor weights for the miR-150, scrambled, and control groups were 0.12 g±0.04, 0.24 g±0.05, and 0.27 g±0.05, respectively (Figures 3f and g). In addition, immunohistochemistrical analysis of the proliferative maker Ki67 and the transferase-mediated deoxyuridine triphosphate-biotin nick end labeling (TUNEL) assay were performed in the tumor sections, and the results showed decreased cell proliferative rate and increased apoptosis in the mice treated with miR-150 oligonucleotides (Figure 3h). The *in vivo* results support the tumor suppression function of miR-150 in leukemia.

### Effects of miR-150 on gene transcriptions and various biological processes

**Figure 4:**
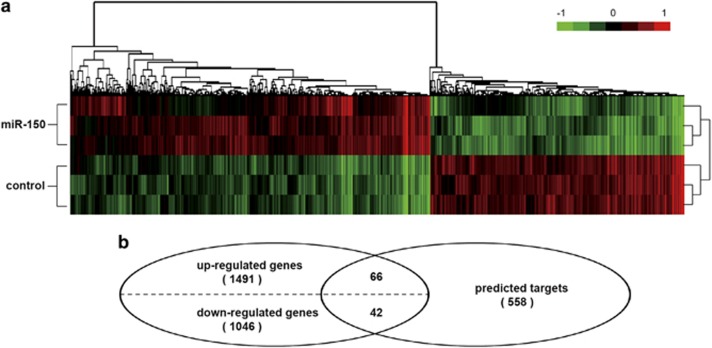
Gene expression profile of miR-150 overexpression. (**a**) Gene tree represented on the top and heat map underneath of microarray analysis (*n*=3). (**b**) Venn diagram showing the common miR-150-regulated transcripts between microarray mRNA profiling and predicted targets

To delineate the molecular basis of miR-150 tumor suppression roles in leukemia, we performed gene expression analysis in K562 cells transfected with miR-150 or the empty lentiviral vector control. The microarray analysis showed a different pattern of gene expression between miR-150 and empty lentivirus-transfected cells (Figure 4a; class comparison, *P*<0.03, false discovery rate (FDR)<0.05). After ectopic transfection of miR-150, 1792 probes (1491 genes) were significantly upregulated and 1266 probes (1046 genes) downregulated (Figure 4b). These differentially expressed genes were analyzed with the gene ontology (GO) as well as pathway browser tools to identify their biological functions (Supplementary Tables S1 and S2). These results show that the expression of miR-150 directly or indirectly affects many biological processes. In particular, many affected genes are involved in the regulation of transcription, metabolic process, regulation of apoptosis, and intracellular signaling cascades.

### Validation of predicted targets

**Figure 5:**
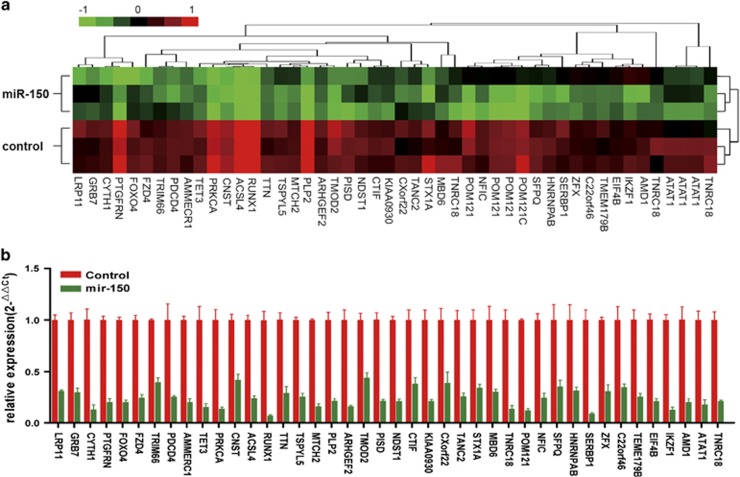
Validation of the microarray analysis. (**a**) Gene tree represented on the top and heat map underneath of miR-150 predicted downregulated targets (*n*=3). (**b**) qRT-PCR validation of the miR-150 downregulated targets identified by microarray. The results were normalized to control cells. RNA levels were normalized with 18s rRNA. Data are the average of three independent experiments±S.D. (miR-150 *versus* control, *P*<0.001)

**Figure 6:**
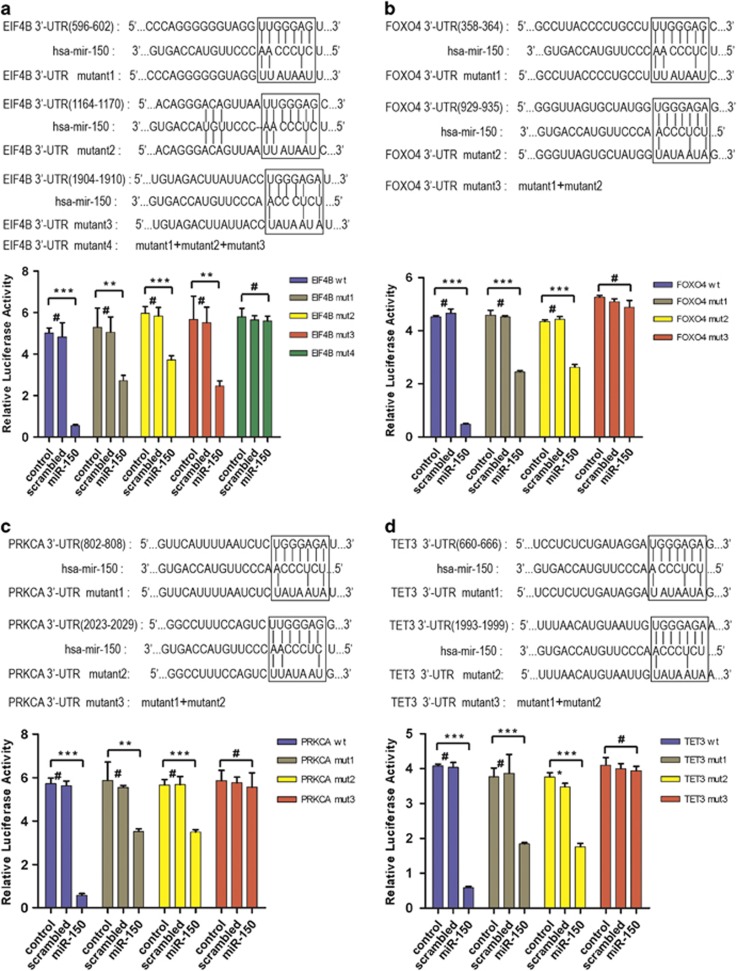
Validation of four of the targets by luciferase assays. Predicted interaction sites of miR-150 in the (**a**) *EIF4B*, (**b**) *FOXO4*, (**c**) *PRKCA*, and (**d**) *TET3* 3′-UTR mRNA, and their site-directed mutations were described above the illustrations. Luciferase activities were determined at 24 h by normalizing firefly to renilla luciferase activity, and expressed as mean values±S.D. from three independent experiments in triplicate. *P<0.05; **P<0.01; ***P<0.001; #, no significance

Among the miR-150 downregulated genes, we identified 42 of them that overlapped with a subset of the 558 miR-150 target genes (Figures 4b and 5a) predicted according to TargetScan.^17^ We validated the microarray results (Figure 5a) by qRT-PCR (*P*<0.001, Figure 5b). In addition, we performed the luciferase reporter assay on four of the downregulated targets (Figure 6). The 3′-untransted region (3′-UTR) of *EIF4B*, *FOXO4*, *PRKCA*, and *TET3* predicted to interact with miR-150 were cloned into a pMIR reporter luciferase vector and co-transfected with miR-150 into 293T cells. A marked reduction in the luciferase activity was observed when *EIF4B*, *FOXO4*, *PRKCA*, or *TET3* construct was co-transfected with synthetic miR-150, but not with the scrambled oligonucleotides (Figures 6a–d). According to TargetScan, there are three predicted interaction sites for miR-150 in the EIF4B 3′-UTR and two sites are predicted to interact with miR-150 in the *FOXO4*, *PRKCA*, and *TET3* 3′-UTR. Next, the studies were done with random mutations in the recognition sequence in 3′-UTR, as described in the upper panel of the illustration (Figure 6), which resulted in impairment of the reporter inhibition by miR-150. As expected, the observed luciferase activity reduction was abrogated when all of the miR-150 interaction sites in 3′-UTR of these targets were mutated. As expected, markedly decreases in the expression of all four target proteins were detected both *in vitro* cultured cells and in xenograft tumors of the miR-150-treated group (Supplementary Figure S4). These results demonstrate that miR-150 regulates *EIF4B*, *FOXO4*, *PRKCA*, and *TET3* expression through binding with their 3′-UTR.

### Functional characterization of miR-150 targets

To further characterize the potential molecular and biological function of miR-150, we carried out GO and pathway analysis to annotate and categorize a subset of its regulated genes (Table 1). They are implicated in small-molecule metabolic process (*POM121*, *PISD*, *STX1A*, *AMD1*, *PRKCA*, *NDST1*, and *ACSL4*), transcriptional regulation (*FOXO4*, *IKZF1*, *TET3*, *NFIC*, *RUNX1*, *EIF4B*, and *CTIF*), RNA metabolic process (*EIF4B*, *PRKCA*, and *PDCD4*), proteoglycan synthesis in cancer *(PDCD4*, *PRKCA*, *FZD4*, and *EIF4B*), mTOR signaling pathway (*PRKCA* and *EIF4B*), and Wnt signaling pathway (*FZD4* and *PRKCA*). These results suggest that miR-150 suppresses leukemia by controlling genes in multiple biological processes and signaling cascades.

**Figure 7:**
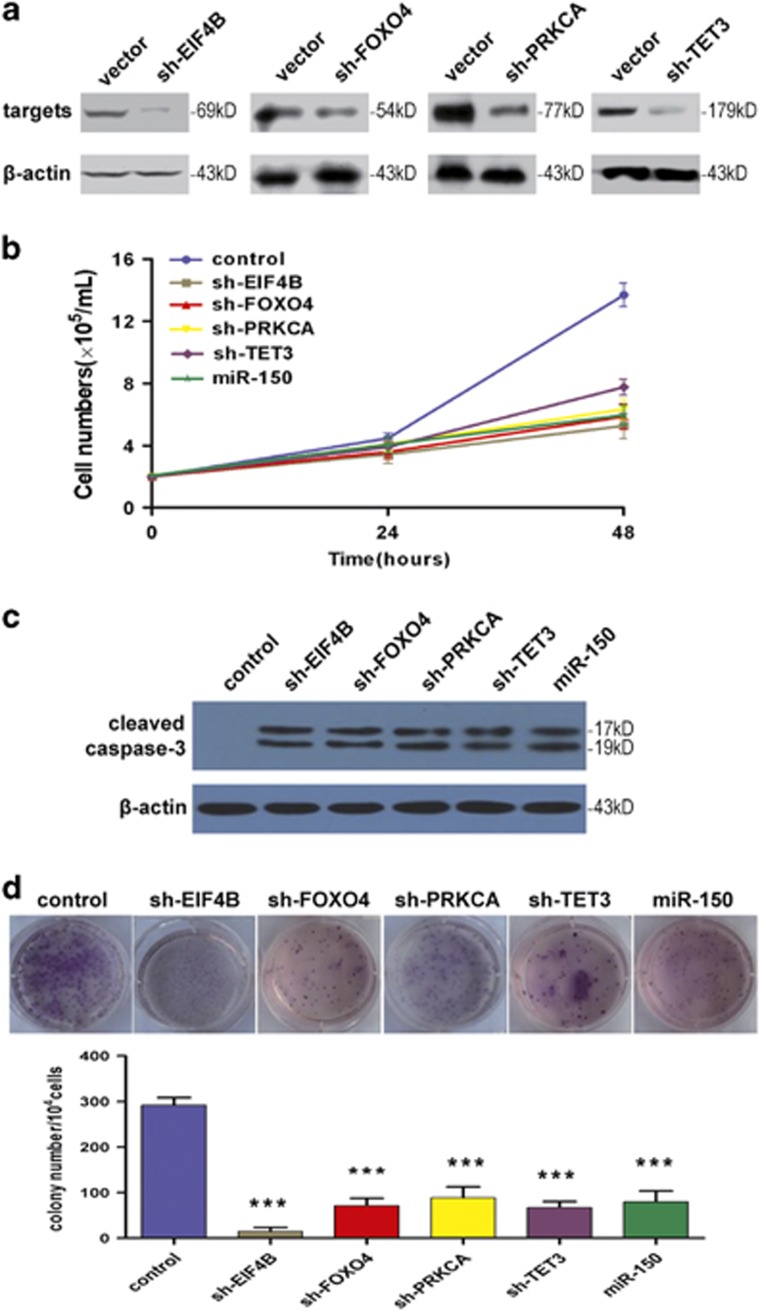
Knockdown of miR-150 targets (*EIF4B*, *FOXO4*, *PRKCA*, and *TET3*) inhibits leukemia cell growth. (**a**) Western blot analyses showing the efficiency of shRNA for EIF4B, FOXO4, PRKCA, and TET3, respectively. *β*-Actin was used as the loading control. (**b**) Cell growth curves of K562 cells infected *in vitro* with miR-150 or shRNA against its targets indicated. (**c**) Apoptosis activation measuring by the level of cleaved caspase-3 with western blot. (**d**) Representative images and statistics of soft agar colony formation assay. K562 cells were transfected with the indicated shRNA or an empty vector as control. Cells were then cultured in methylcellulose. Colonies were counted after 14 days. All data are represented as mean values±S.D. (*n*=3). ***P<0.001 for each shRNA group compared with control

We next investigated whether these targets indeed are involved in miR-150-mediated tumor suppression in leukemia. We used specific short hairpin RNA (shRNA) to knockdown one of four representative miR-150 targets (*EIF4B*, *FOXO4*, *PRKCA*, or *TET3*). Three shRNA sequences were designed to against each target. One potent and specific shRNA was selected in subsequent experiments. As shown in Figure 7a, efficient knockdown was achieved for each target protein. Importantly, suppressing the expression of any of the four targets inhibited leukemia cells growth as well as induced apoptosis in consistent with the effect of miR-150 (Figures 7b and c). Furthermore, one of the transformed properties of human K562 cells is their ability to grow in semi-solid media. K562 cells were transduced with these shRNAs or miR-150. Knockdown of EIF4B, FOXO4, PRKCA, or TET3 significantly decreased the colony formation of K562 cells, similar as the effect seen in the miR-150-overexpression group (Figure 7d). These results support that ectopic expression of miR-150 suppresses leukemia by simultaneously inhibiting multiple oncogenes.

## Discussion

Deregulation of several miRNAs has been linked to leukemogenesis and miRNAs may therefore serve as promising therapeutic targets for leukemia.^3, 18^ However, the precise mechanisms of action of many miRNAs are largely unknown. In the present study, we show that miR-150 expression is markedly downregulated in most human ALL, AML, and CML specimens tested compared with healthy controls. We demonstrated that miR-150 exerts a tumor suppressor function *in vitro* by inhibiting proliferation and inducing apoptosis in both leukemia cell lines and primary AML cells, and *in vivo* by reducing the growth of human leukemia engraftments in nude mice. Furthermore, we investigated the molecular bases of miR-150 tumor suppressor function and identified the pathways regulated by miR-150 using microarray analysis in conjunction with TargetScan, a widely used methodology for the identification of miRNA targets.^19^ Using this approach, we identified 42 potential miR-150 target genes that were confirmed by qRT-PCR and some of them were further validated by luciferase reporter assays. GO and pathway analyses revealed that these targets are significantly enriched in various important biological processes such as cell metabolic process, transcriptional regulation, as well as Wnt and mTOR signaling pathways.

Importantly, most of these 42 genes were previously uncharacterized targets for miR-150. Of note, Jiang *et al.*^12^ recently showed that miR-150 inhibits MLL/AF9-mediated leukemia transformation through targeting *MYB* and *FLT3*. Another groups identified *CBL* and *EGR2* as bona fide targets of miR-150 in MLL/AF9-transformed leukemia,^13^ and loss of CBL sensitizes leukemia to chemotherapy.^20^ The differences between our findings and the previous reports could be explained in part by the differences in genetic backgrounds and methodologies used. Although most of the identified targets are involved in the cancer pathway, many have been shown to be essential but not sufficient individually for leukemia transformation.^21, 22, 23^ We, therefore, speculate that miR-150 suppresses leukemia transformation through regulating multiple genes that involve in and coordinate several biological processes and pathways.^24^

Protein kinase C*-α* (PKC*α*), one of PKC isoforms, has long been recognized as a regulator of tumor growth in many kinds of cancers.^25, 26, 27, 28, 29^ There has been a great interest in targeting this kinase for cancer therapy. For example, targeting PKC*α*-mediated signal transduction pathway induces cell killing in AML cells by inhibiting Bcl-2 phosphorylation and blocking ERK activation.^30^ Herein, we showed that forced expression of miR-150 significantly repressed endogenous expression of PKC*α*, and luciferase reporter/mutagenesis assays confirmed that *PRKCA* is a transcriptional target of miR-150. Furthermore, we showed that knockdown PKC*α* expression inhibited leukemia cell growth, supporting that PKC*α* is an essential target and has critical roles in miR-150-mediated antagonism to leukemia.

The forkhead box O (FOXO) family of proteins are transcription factors and share paradoxical roles in cancer progression.^31, 32^ On one hand, FOXOs are tumor suppressors and inactivated by major oncogenic signals such as the phosphatidylinositol-3 kinase (PI3K) and MAP kinases. On the other hand, active FOXOs maintain leukemia stem cell quiescence, induce drug resistance, and contribute to leukemogenesis. FOXO4 has been identified as a fusion protein with MLL in a subset of AML and ALL with a t(X;11)(q13;q23).^33, 34, 35, 36^ In the MLL/FOXO4 fusion, the conserved transactivation domain seems to be critical for its full oncogenicity in hematopoietic progenitors.^34, 35^ Interestingly, MLL/FOXO4 was shown to antagonize the transcriptional activity of endogenous FOXO3 and repress its ability to induce apoptosis, suggesting distinct roles for FOXO family members.^34^ FOXOs were found to be active in 40% of AML patient samples regardless of genetic subtype.^37^ In a murine model of AML induced by the MLL/AF9 fusion gene, leukemia-initiating cells show a high activity of FOXOs.^37^ In these cells, deletion of FOXO1/3/4 markedly diminishes leukemia-initiating cells function and improves animal survival. Collectively, these studies suggest that FOXO4 acts as a pro-tumor factor in leukemia. The expression and function of FOXOs are regulated by some oncogenic signaling pathways, such as PI3K, ERK, or IKK pathway.^32^ Moreover, several miRNAs have been identified as regulators of FOXO expressions in different cancer types.^32^ Here we substantiated that FOXO4 is a bona fide target of miR-150. Consistent with its oncogenic role in leukemia, we found that depletion of FOXO4 expression reduces leukemic cells growth.

In addition, we identified *EIF4B* and *TET3* as important targets of miR-150. eIF4B has important roles in ABL-induced leukemia and in the pathogenesis of diffuse large B-cell lymphoma.^38, 39, 40^ In ABL-transformed leukemia cell line K562, eIF4B is activated and integrates the signals from the Pim and PI3K/Akt/mTOR pathway.^39^ In contrast, cells derived from eIF4B knockdown transgenic mice are less susceptible to ABL transformation.^38^ Similar to these observations, we found that silencing eIF4B by shRNA reduces the colony formation ability of K562 cells. The ten-eleven-translocation (TET) family dioxygenases function as epigenetic modulators by oxidizing 5-methylcytosine to 5-hydroxymethylcytosin (5hmC). Among its three members, TET2 acts as a tumor suppressor and is mutated in a wide range of hematological malignancies.^41, 42^ Loss of function analyses revealed that TET2 and TET3 have redundant roles in hematopoietic system and can compensate for one another.^42^ A miR-15b–TET3–cyclin D1 axis is important for neocortical development, where miR-15b reduces TET3 expression and 5hmC levels and regulates neural progenitor proliferation through epigenetic control of cyclin D1 expression.^43^ Whether miR-150–TET3 functions in epigenetic regulation in leukemia is under investigation.

Collectively, our results help to delineate a complex signaling network mediated by miR-150 in leukemia. The study not only broadens our understanding of the complex mechanisms underlying the pathogenesis of miR-150 deregulation in leukemia but also provides a rationale to target miR-150 for the treatment of leukemia.

## Materials and Methods

### Patient samples and cell culture

Bone marrow from leukemia patients and healthy donors was obtained after informed consent in accordance with the guidelines of the first hospital affiliated to Xiamen university, and the study protocols were approved by the Ethics Committee. Human leukemia cell lines K562, Kasumi-1, and THP-1, and human embryonic kidney cell line 293T were used in this study and maintained, respectively, in PRMI 1640 and DMEM medium (Gibco, Carlsbad, CA, USA) supplemented with 10% heat-inactivated fetal bovine serum, l-glutamine, and antibiotics at 37 °C in 5% CO_2_.

### Real-time qRT-PCR

To evaluate miR-150 expression in bone marrow mononuclear cells, real-time qRT-PCR for miRNA was performed. Mononuclear cells were isolated by Ficoll Hypaque (Cedarlane, Burlington, ON, Canada) density gradient centrifugation as described.^44^ Total RNA was extracted from mononuclear cells using a miRcute miRNA isolation kit (Tiangen Biotech, Beijing, China) according to the manufacturer's protocol. The miR-150- and U6 (as an internal control)-specific cDNA were synthesized from total RNA using gene-specific primers according to the miRcute miRNA qRT-PCR protocol (Tiangen Biotech). The primers used for miR-150 (CD201-0014) and U6 snRNA (CD201-0145) were also obtained from Tiangen Biotech. Relative quantification of target miRNA expression was evaluated using the comparative cycle threshold (CT) method. The data were presented as the relative quantity of target miRNA, normalized with respect to U6. Each sample was examined in triplicate. Mean normalized gene expression±S.D. was calculated from independent experiments.

### Lentivirus production and infection

The miR-150-overexpressing vector is a feline immunodeficiency virus-based construct (GeneCopoeia, Guanzhou, China) consisting of the stem loop structure of miR-150 and its flanking genomic sequence cloned into the pEZX-MR plasmid. Pseudoviral particles were generated using the Lenti-Pac HIV expression packaging kit (GeneCopoeia), according to the manufacturer's instructions. For the transduction, leukemic cells were infected with miR-150 lentivirus with an efficiency of ~50% as measured by GFP expression under an Olympus digital fluorescence microscope (Olympus, Tokyo, Japan). miR-150 overexpression was confirmed by qRT-PCR. Empty lentivirus was used as a control for the experiments.

### Cell growth and apoptosis analysis

Cells were collected and counted at 48-h intervals using trypan blue staining. Cell viability was also validated using the CCK8 test. Apoptotic cell death was analyzed by measuring externalized phosphatidyl serine with an Annexin V/7-AAD staining kit (BD, Franklin Lakes, NC, USA).

### Mouse xenograft model studies

Animal studies were performed according to the Xiamen University institutional guidelines. K562 cells were transfected *in vitro* with pseudoviral particles expressing miR-150. Cells transfected with the same empty lentivirus served as tumorigenic controls. At 24 h after the transfection, a total of 5 × 10^6^ viable cells were injected subcutaneously into the right flanks of 5-week-old female nude mice. The tumor size was measured every 3 days. At the 34th day, the mice were killed and tumors were weighted. Tumor volumes were calculated using the formula: *V* (mm^3^)=*A* × *B*^2^/2, where *A* is the longest and *B* the shortest diameter. For the inhibition experiment, untreated cells were injected subcutaneously into the mice. When the tumor reached ~50 mm^3^, 5 *μ*g of synthetic miR-150 or scrambled oligonucleotides diluted in Lipofectamine (Invitrogen, Carlsbad, CA, USA) solution was injected directly into the tumors at days 1, 4, 7, and 10. The mice were killed and tumors were weighted at day 16.

### Transcriptional profiling and data analysis

Total RNA was extracted and purified from K562 cells transfected with miR-150 and the empty vector using the TRizol and RNeasy kit (Qiagen, Hilden, Germany). Samples were analyzed using GeneChip Human Gene 1.0ST Array (Affymetrix, Santa Clara, CA, USA) with technical support from Shanghai Gminix-Cloud Biotechnology Corporation (Shanghai, China). The differentially expressed genes were selected as having more than twofold difference between their geometrical mean expression in the compared groups and a statistically significant *P*-value (<0.05) by analysis of variance (ANOVA). The GO analysis on differentially expressed genes was performed with the DAVID (database for annotation, visualization and integrated discovery) functional annotation tool using a *P*<0.05 to define statistically enriched GO categories.^45^ Pathway analysis was used to determine the significant pathway of the differential genes according to Kyoto Encyclopedia of Genes and Genomes Database (http://www.genome.jp/kegg/). The analysis of miRNA-predicted targets was determined using the algorithms TargetScan 6.2 (http://www.targetscan.org).

### Luciferase reporter and mutagenesis assay

Luciferase reporter and mutagenesis assay were conducted as described previously with modifications.^44^ In brief, for luciferase reporter experiments, a 3′-UTR segment of target gene was amplified by PCR from human cDNA and inserted into the pMIR reporter luciferase vector with CMV promoter (Promega, Madison, WI, USA), using the *MIu*I and *Pme*l site immediately downstream from the stop codon of luciferase. The sets of primers used to generate specific fragments are shown in Supplementary Data (Supplementary Table S3).

Luciferase assays were performed using the Dual-Luciferase reporter system (Promega). The transfection efficiency was monitored by co-transfected with pRL, an expression vector of renilla reniformis luciferase. In brief, 293T cells were co-transfected with 100 ng of the firefly luciferase report vector and 50 ng of pRL in 24-well plates using Lipofectamine 2000 reagent following the manufacturer's protocol. For each well, 100 nM (final concentration) of synthetic miR-150 or scrambled oligonucleotides was used. Cells were then collected and lysed in lysis buffer supplied by the manufacturer, followed by the measurement of firefly and renilla luciferase activities on luminometer, 24 h after the transfection. The relative firefly luciferase activities were calculated by normalizing transfection efficiency according to the renilla luciferase activities.

### RNA interference and clonogenicity assay

shRNA against *EIF4B*, *FOXO4*, *PRKCA*, or *TET3* was subcloned into a shRNA expression vector psiLv-H1 (GeneCopoeia). The sequences of the shRNAs used in the experiment are provided in Supplementary Data (Supplementary Table S4). Lentivirus-containing shRNA and psilv-H1 backbone were produced using the Lenti-Pac HIV expression packaging kit (GeneCopoeia), following the manufacturer's instructions. After 48 h, the supernatant was collected and used for transduction. The transduction efficiency was measured by a fluorescence microscope, and the efficiency of knockdown was determined by western blot. For clonogenicity assay, K562 cells were transfected with the shRNAs or miR-150. Following 48-h transfection, cells were plated in 0.9% methylcellulose media for colony formation. After 2 weeks, colonies were fixed with methanol, stained with crystal violet, photographed, and counted.

### Western blot

Protein extracts were resolved by SDS-polyacrylamide gel electrophoresis, transferred to polyvinylidene fluoride membranes (Millipore, Bedford, MA, USA). The membranes were probed with primary antibodies, and then with peroxidase-conjugated secondary antibody (Beyotime, Suzhou, China), followed by visualization using chemiluminescence assay (Pierce, Rockford, IL, USA). Antibodies against EIF4B, FOXO4, PRKCA (Abcam, Cambridge, UK), TET3 (Abnova, Taipei, Taiwan), cleaved caspase-3 (Asp175; Cell Signaling Technology, Danvers, MA, USA), and *β*-actin (Santa Cruz Biotechnology, Santa Cruz, CA, USA) were used in this study.

### Histological examination

Tumor tissues were fixed in 10% formalin and embedded in paraffin. Four-micron tissue sections was prepared, deparaffinized, dehydrated, and then stained with hematoxylin and eosin. Immunohistochemical staining was performed with the anti-human Ki67 (Santa Cruz Biotechnology) using standarded methods. TUNEL staining was assessed using the *In Situ* Cell Death Detection kit (Fluorescein; Roche, Mannheim, Germany) according to the manufacture's instructions.

### Statistical analysis

Data are presented as mean±S.D. The differences between the means were tested by the Student's *t*-test or one-way ANOVA. *P*<0.05 was defined as statistically significant.

## Figures and Tables

**Table 1:** Functional annotation of miR-150-targeted genes

*Functional annotation*	*Target gene*	*%*^a^	P-*value*
*GO category*			
Small-molecule metabolic process	*POM121*, *PISD*, *STX1A*, *AMD1*, *PRKCA*, *NDST1*, *ACSL4*	16.67	0.001
Fibroblast growth factor receptor signaling pathway	*FOXO4*, *PRKCA*, *NDST1*	7.14	0.001
Positive regulation of transcription from RNA polymerase II promoter	*FOXO4*, *IKZF1*, *TET3*, *NFIC*, *RUNX1*	11.9	0.002
Regulation of translational initiation	*EIF4B*, *CTIF*	4.76	0.002
Epidermal growth factor receptor signaling pathway	*GRB7*, *FOXO4*, *PRKCA*	7.14	0.003
Polysaccharide biosynthetic process	*NDST1*	2.38	0.004
*S*-adenosylmethioninamine biosynthetic process	*AMD1*	2.38	0.004
Negative regulation of prostaglandin secretion	*ACSL4*	2.38	0.004
Forward locomotion	*TTN*	2.38	0.004
DNA demethylation of male pronucleus	*TET3*	2.38	0.004
Positive regulation of neutrophil differentiation	*IKZF1*	2.38	0.004
Regulation of vasculature	*FZD4*	2.38	0.004
Alpha-tubulin acetylation	*ATAT1*	2.38	0.004
Positive regulation of dense core granule biogenesis	*PRKCA*	2.38	0.004
*In utero* embryonic development	*TTN*, *RUNX1*, *TANC2*	7.14	0.004
mRNA transport	*POM121*, *POM121C*	4.76	0.005
Forebrain development	*NDST1*, *IKZF1*	4.76	0.006
Histone H3-T6 phosphorylation	*PRKCA*	2.38	0.008
Progesterone secretion	*FZD4*	2.38	0.008
Positive regulation of Golgi to plasma membrane protein transport	*CNST*	2.38	0.008
RNA metabolic process	*EIF4B*, *PRKCA*, *PDCD4*	7.14	0.008
Neurotrophin TRK receptor signaling pathway	*PRKCA*, *ARHGEF2*, *FOXO4*	7.14	0.009
Positive regulation of angiogenesis	*PRKCA*, *RUNX1*	4.76	0.011
Apoptotic signaling pathway	*PRKCA*, *ARHGEF2*	4.76	0.016
			
*Pathway*			
Proteoglycans in cancer	*PDCD4*, *PRKCA*, *FZD4*, *EIF4B*	9.52	0.0002
RNA transport	*POM121C*, *POM121*, *EIF4B*	7.14	0.002
Pathogenic *Escherichia coli* infection	*ARHGEF2*, *PRKCA*	4.76	0.005
mTOR signaling pathway	*PRKCA*, *EIF4B*	4.76	0.006
Amphetamine addiction	*PRKCA*, *STX1A*	4.76	0.008
Insulin secretion	*PRKCA*, *STX1A*	4.76	0.012
Pathways in cancer	*FZD4*, *RUNX1*, *PRKCA*	7.14	0.015
Melanogenesis	*FZD4*, *PRKCA*	4.76	0.017
Wnt signaling pathway	*FZD4*, *PRKCA*	4.76	0.033

a From total 42.

## Abbreviations

3′-UTR: 3′-untransted region
AML: acute myeloid leukemia
ALL: acute lymphoblastic leukemia
Ara-C: cytarabine
CML: chronic myeloid leukemia
HSC: hematopoietic stem cell
PKC: protein kinase C
TUNEL: transferase-mediated deoxyuridine triphosphate-biotin nick end labeling
