# Supplementary material for: miR-150 exerts antileukemia activity in vitro and in vivo through regulating genes in multiple pathways
Source: Cell Death Dis. 2016 Sep 22;7(9):e2371–. doi: 10.1038/cddis.2016.256 (PMC5059860; doi:10.1038/cddis.2016.256)
Supplement: Supplementary Information [file cddis2016256x1.doc]

**Supplementary Figure Legends**

**Figure S1** **miR-150 inhibits the cell viability in leukemia cell lines.** Leukemia cell lines were transfected with miR-150 or empty vector and the cell viability was detected by CCK8 test after 24 hours and 48 hours. (a) K562, (b) Kasumi-1, (c) THP-1. Data are mean ± SD of triplicates assays. *，*P* ＜ 0.05,**, *P* ＜ 0.01; #, no significance.

**Figure S2 miR-150 sensitizes the cytotoxicity of Ara-C.** Cells were transfected with miR-150 or empty vector in the presence or absence of 5 μM of Ara-C, then cell viability was detected by CCK8 test after 24 hours and 48 hours. (a) K562, (b) Kasumi-1, (c) THP-1. The results were shown as mean ± SD of three separated experiments. *, *P* ＜ 0.05, **, *P* ＜ 0.01, ***, *P* ＜ 0.001; #, no significance.

**Figure S3 The level of miR-150 in the tumor tissues.** (a) miR-150 expression was analyzed by qRT-PCR in K562 cells overexpressing miR-150 and its counterpart control cells before inoculation into mice. At the 34^th^ day after xenograft, the mice were sacrificed and the level of miR-150 in tumor tissue was detected by the same method (n=8). (b) The miR-150 level in tumor lumps from mice with different treatments at the end of experiment (n=8). Data are mean ± SD. ***, *P* ＜ 0.001. #, no significance.

**Figure S4 Western blot assays of the target proteins.** EIF4B, FOXO4, PRKCA, and TET3 protein levels were detected both *in vitro* cultured cells and *in vivo* tumor tissues. β-actin was used as a loading control for the western blot.
