# Supplementary figures and images for: miR-150 exerts antileukemia activity in vitro and in vivo through regulating genes in multiple pathways
Source: Cell Death Dis. 2016 Sep 22;7(9):e2371–. doi: 10.1038/cddis.2016.256 (PMC5059860; doi:10.1038/cddis.2016.256)

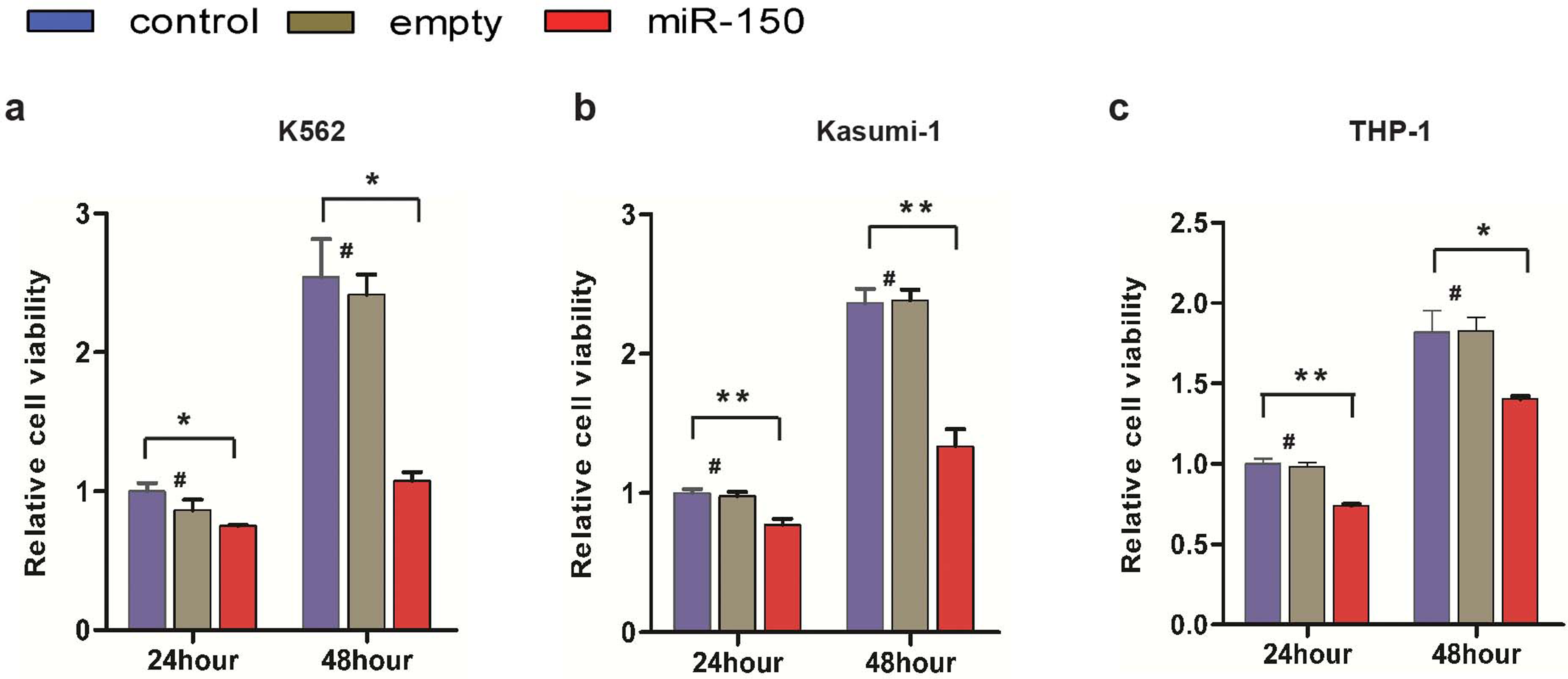

Supplement: Supplementary Figure 1 [file cddis2016256x2.tif]

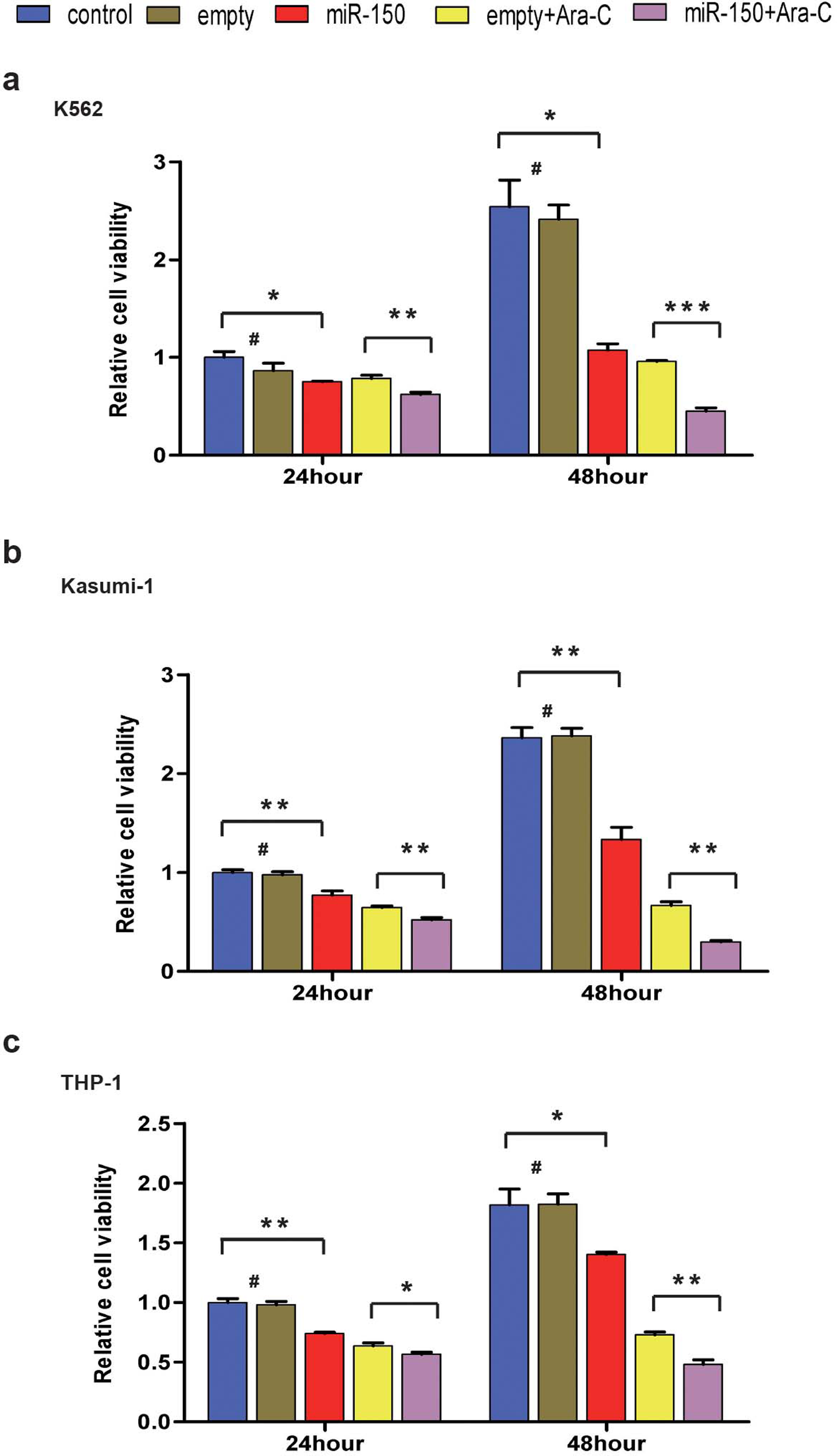

Supplement: Supplementary Figure 2 [file cddis2016256x3.tif]

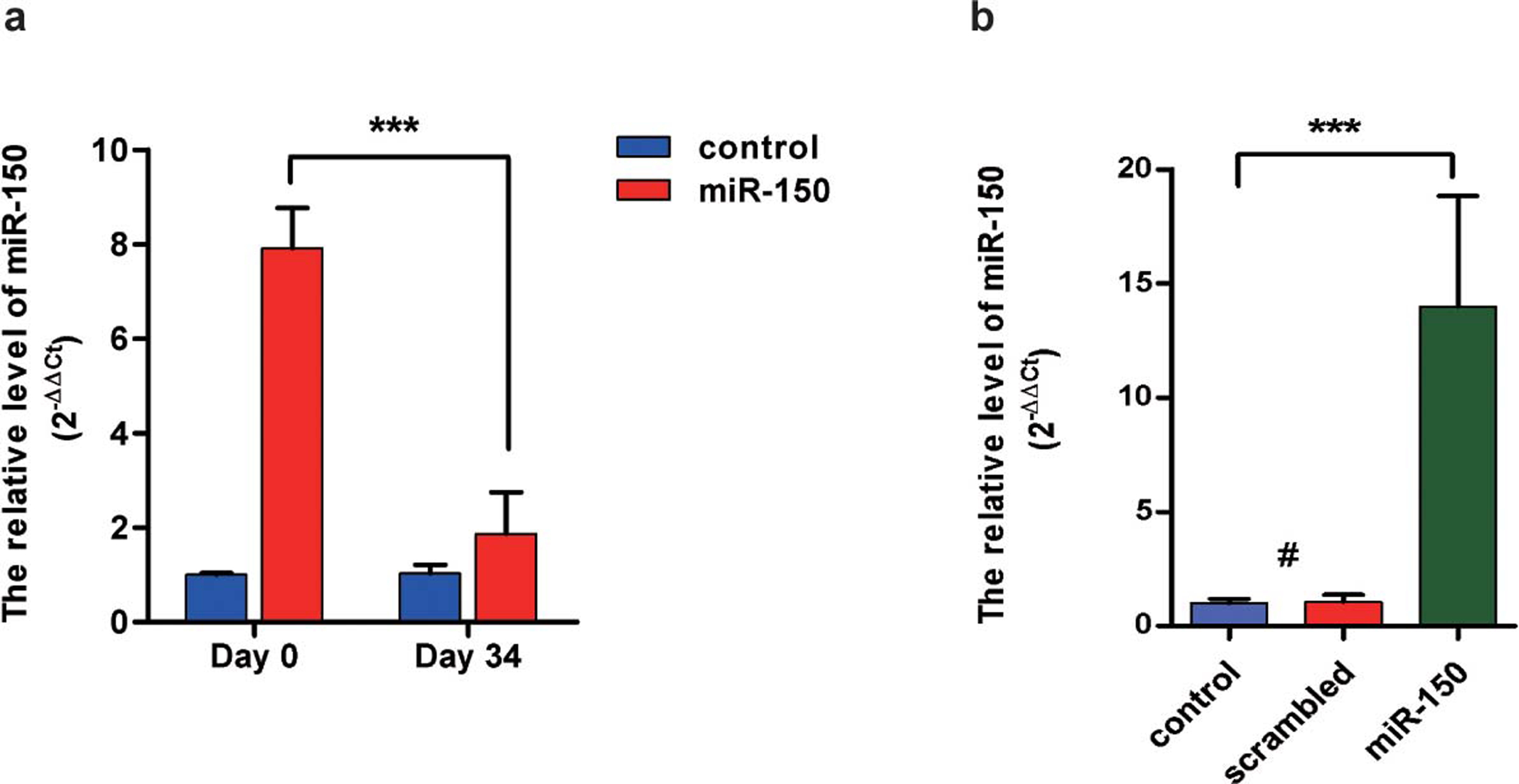

Supplement: Supplementary Figure 3 [file cddis2016256x4.tif]

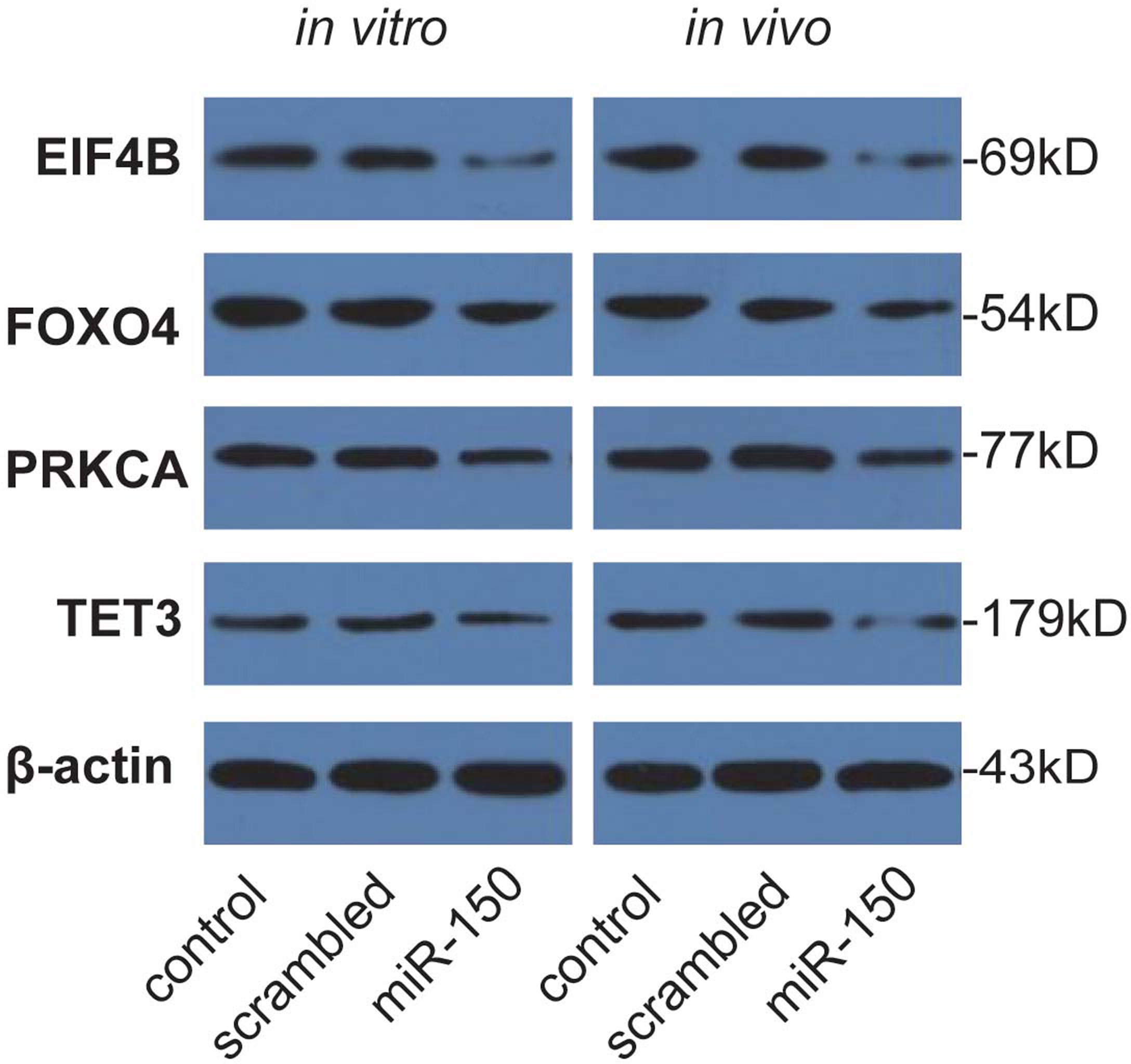

Supplement: Supplementary Figure 4 [file cddis2016256x5.tif]
