# Supplementary material for: miR-150 exerts antileukemia activity in vitro and in vivo through regulating genes in multiple pathways
Source: Cell Death Dis. 2016 Sep 22;7(9):e2371–. doi: 10.1038/cddis.2016.256 (PMC5059860; doi:10.1038/cddis.2016.256)
Supplement: Supplementary Table 1 [file cddis2016256x6.doc]

**Table S1 Functional annotation of down-regulated genes**

| **Functional annotation** | **Count** | **% *** | **P value** |
| --- | --- | --- | --- |
| ***GO category***  small molecule metabolic process  positive regulation of transcription from RNA polymerase II promoter  signal transduction  innate immune response  transcription, DNA-dependent  regulation of transcription from RNA polymerase II promoter  blood coagulation  negative regulation of transcription from RNA polymerase II promoter  apoptotic process  transcription from RNA polymerase II promoter  negative regulation of apoptotic process  gene expression  regulation of transcription, DNA-dependent  metabolic process  neurotrophin TRK receptor signaling pathway  transmembrane transport  positive regulation of transcription, DNA-dependent  ribosome biogenesis  positive regulation of gene expression  protein phosphorylation  phospholipid metabolic process  negative regulation of cell proliferation  cellular nitrogen compound metabolic process  energy reserve metabolic process  apoptotic signaling pathway  cellular amino acid biosynthetic process  intrinsic apoptotic signaling pathway  glucose metabolic process  intracellular signal transduction  response to corticosterone stimulus  platelet activation  carbohydrate metabolic process  insulin receptor signaling pathway  lipid metabolic process  phospholipid biosynthetic process  negative regulation of transcription, DNA-dependent  positive regulation of GTPase activity  epidermal growth factor receptor signaling pathway  Fc-epsilon receptor signaling pathway  spliceosomal snRNP assembly  cell differentiation  triglyceride biosynthetic process  lipid biosynthetic process  fatty acid transport  metanephros development  mitosis  hemopoiesis  transport  ***Pathway***  Metabolic pathways  Proteoglycans in cancer  Acute myeloid leukemia  PI3K-Akt signaling pathway  Fc epsilon RI signaling pathway  MAPK signaling pathway  Focal adhesion  FoxO signaling pathway  Pathways in cancer  Oxytocin signaling pathway  Epstein-Barr virus infection  GnRH signaling pathway  Glioma  Dopaminergic synapse  Inflammatory mediator regulation of TRP channels  HTLV-I infection  Transcriptional misregulation in cancer  ErbB signaling pathway  Carbon metabolism  mTOR signaling pathway  Fc gamma R-mediated phagocytosis  Morphine addiction  Cholinergic synapse  MicroRNAs in cancer  Calcium signaling pathway  Ras signaling pathway  Insulin signaling pathway  RNA transport  B cell receptor signaling pathway  Prolactin signaling pathway  Rap1 signaling pathway  Osteoclast differentiation  Sphingolipid metabolism  Phosphatidylinositol signaling system  Viral carcinogenesis  Thyroid hormone signaling pathway  Estrogen signaling pathway  Neurotrophin signaling pathway  Long-term potentiation  Retrograde endocannabinoid signaling  Amphetamine addiction  Apoptosis  Progesterone-mediated oocyte maturation  Hepatitis B  Regulation of actin cytoskeleton  Gap junction  GABAergic synapse  Oocyte meiosis  Non-small cell lung cancer  Chronic myeloid leukemia  Glycerophospholipid metabolism  Influenza A  Endocytosis  Alcoholism  Circadian entrainment  Wnt signaling pathway  Herpes simplex infection  Endocrine and other factor-regulated calcium reabsorption | 105  60  67  50  93  34  39  46  44  38  37  45  77  53  27  41  35  10  19  35  18  28  19  14  15  7  11  15  27  7  18  24  15  15  7  28  19  17  16  7  25  8  5  5  7  18  9  24  79  25  13  29  13  23  20  16  25  17  19  13  11  15  13  21  17  12  13  10  12  12  13  21  16  18  14  15  10  10  17  13  8  10  16  12  11  12  9  11  9  10  10  13  16  10  10  11  8  9  10  14  15  14  10  12  14  7 | 10.04  5.74  6.40  4.78  8.89  3.25  3.73  4.40  4.21  3.63  3.54  4.30  7.36  5.07  2.58  3.92  3.35  0.96  1.82  3.35  1.72  2.68  1.82  1.34  1.43  0.67  1.05  1.43  2.58  0.67  1.72  2.29  1.43  1.43  0.67  2.68  1.82  1.62  1.54  0.67  2.39  0.76  0.48  0.48  0.67  1.72  0.86  2.29  7.55  2.39  1.24  2.77  1.24  2.20  1.91  1.53  2.39  1.62  1.82  1.24  1.05  1.43  1.24  2.01  1.62  1.15  1.24  0.96  1.15  1.15  1.24  2.01  1.53  1.72  1.34  1.43  0.96  0.96  1.62  1.24  0.76  0.96  1.53  1.15  1.05  1.15  0.86  1.05  0.86  0.96  0.96  1.24  1.53  0.96  0.96  1.05  0.76  0.86  0.96  1.34  1.43  1.34  0.96  1.15  1.34  0.67 | 2.65E-29  2.66E-15  1.42E-14  9.91E-14  3.08E-13  6.06E-13  1.45E-12  1.77E-12  3.23E-12  2.39E-11  3.12E-11  3.88E-11  5.40E-11  1.10E-10  1.29E-10  4.98E-10  3.17E-09  6.50E-09  8.72E-09  2.17E-08  2.23E-08  3.51E-08  5.29E-08  8.49E-08  1.02E-07  1.05E-07  1.09E-07  1.28E-07  8.24E-07  1.19E-06  1.37E-06  1.63E-06  1.91E-06  2.47E-06  3.02E-06  3.22E-06  3.42E-06  3.42E-06  3.83E-06  5.25E-06  7.75E-06  8.45E-06  1.07E-05  1.07E-05  1.10E-05  1.19E-05  1.36E-05  1.49E-05  8.53E-19  5.09E-11  5.16E-10  1.19E-09  7.80E-09  2.34E-08  5.79E-08  7.82E-08  1.23E-07  1.68E-07  2.20E-07  2.41E-07  3.75E-07  4.19E-07  5.85E-07  7.45E-07  9.48E-07  1.15E-06  1.47E-06  1.67E-06  1.68E-06  2.13E-06  2.78E-06  5.16E-06  5.16E-06  6.06E-06  6.34E-06  7.69E-06  9.49E-06  9.49E-06  1.08E-05  1.62E-05  2.05E-05  2.81E-05  2.97E-05  2.97E-05  3.07E-05  3.24E-05  4.01E-05  4.08E-05  4.53E-05  4.83E-05  4.82E-05  4.91E-05  5.08E-05  6.5E-05  7.24E-05  7.66E-05  7.87E-05  8.14E-05  8.79E-05  0.0001  0.0001  0.0001  0.0001  0.0001  0.0002  0.0002 |

*from total 1046
