# Supplementary material for: miR-150 exerts antileukemia activity in vitro and in vivo through regulating genes in multiple pathways
Source: Cell Death Dis. 2016 Sep 22;7(9):e2371–. doi: 10.1038/cddis.2016.256 (PMC5059860; doi:10.1038/cddis.2016.256)
Supplement: Supplementary Table 2 [file cddis2016256x7.doc]

**Table S2 Functional annotation of up-regulated genes**

| **Functional annotation** | **Count** | **% *** | **P value** |
| --- | --- | --- | --- |
| ***GO category***  small molecule metabolic process  transcription, DNA-dependent  signal transduction  regulation of transcription, DNA-dependent  microtubule cytoskeleton organization  blood coagulation  small GTPase mediated signal transduction  protein transport  metabolic process  transmembrane transport  cellular protein metabolic process  positive regulation of transcription from RNA polymerase II promoter  insulin receptor signaling pathway  negative regulation of transcription, DNA-dependent  negative regulation of transcription from RNA polymerase II promoter  platelet activation  regulation of small GTPase mediated signal transduction  oxidation-reduction process  cell migration  cytokine-mediated signaling pathway  cilium assembly  GTP catabolic process  protein phosphorylation  substrate adhesion-dependent cell spreading  axon guidance  post-embryonic development  negative regulation of cell proliferation  cell adhesion  viral reproduction  innate immune response  transferrin transport  interaction with host  ion transmembrane transport  phosphatidylinositol biosynthetic process  fat cell differentiation  positive regulation of transcription, DNA-dependent  intracellular signal transduction  positive regulation of GTPase activity  brain development  visual perception  ATP hydrolysis coupled proton transport  phagosome maturation  epidermal growth factor receptor signaling pathway  protein ubiquitination  ***Pathway***  Metabolic pathways  Vibrio cholerae infection  Phagosome  Regulation of actin cytoskeleton  Collecting duct acid secretion  Oxidative phosphorylation  Phosphatidylinositol signaling system  Lysosome  Proteoglycans in cancer  Epithelial cell signaling in Helicobacter pylori infection  Inflammatory mediator regulation of TRP channels  Inositol phosphate metabolism  Synaptic vesicle cycle  Amoebiasis  Pathways in cancer  mTOR signaling pathway  FoxO signaling pathway  VEGF signaling pathway  Alzheimer's disease | 112  134  92  111  18  46  36  40  67  51  46  62  22  41  49  25  21  47  21  27  17  27  41  10  30  14  32  36  41  46  9  9  23  11  11  37  32  23  19  20  8  9  20  28  96  14  22  25  9  18  14  17  23  12  14  11  11  14  26  10  15  10  17 | 7.51  8.99  6.17  7.44  1.21  3.08  2.41  2.68  4.49  3.42  3.08  4.16  1.48  2.75  3.29  1.68  1.41  3.15  1.41  1.81  1.14  1.81  2.75  0.67  2.01  0.94  2.15  2.41  2.75  3.08  0.60  0.60  1.54  0.74  0.74  2.48  2.15  1.54  1.27  1.34  0.54  0.60  1.34  1.88  6.44  0.94  1.48  1.68  0.60  1.21  0.94  1.14  1.54  0.80  0.94  0.74  0.74  0.94  1.74  0.67  1.01  0.67  1.14 | 9.70E-21  2.80E-19  9.07E-19  6.42E-16  5.84E-12  7.97E-12  1.14E-11  3.44E-11  5.69E-11  3.43E-10  4.18E-10  1.36E-09  1.89E-09  3.86E-09  7.53E-09  1.04E-08  2.59E-08  2.67E-08  5.18E-08  6.86E-08  9.27E-08  1.41E-07  2.59E-07  3.34E-07  3.90E-07  4.23E-07  5.08E-07  6.14E-07  6.26E-07  1.26E-06  1.378E-06  1.82E-06  2.53E-06  3.09E-06  3.09E-06  3.17E-06  3.64E-06  3.93E-06  4.17E-06  5.07E-06  5.67E-06  6.43E-06  7.00E-06  7.33E-06  5.59E-18  1.58E-09  7.65E-09  2.78E-08  1.96E-07  3.45E-07  4.23E-07  5.08E-07  1.17E-06  2.70E-06  5.37E-06  6.28E-06  8.77E-06  1.72E-05  2.44E-05  3.90E-05  4.01E-05  4.54E-05  4.56E-05 |

*from total 1491
