# Supplementary material for: miR-150 exerts antileukemia activity in vitro and in vivo through regulating genes in multiple pathways
Source: Cell Death Dis. 2016 Sep 22;7(9):e2371–. doi: 10.1038/cddis.2016.256 (PMC5059860; doi:10.1038/cddis.2016.256)
Supplement: Supplementary Table 3 [file cddis2016256x8.doc]

**Table S3 Primer sequences used for Luciferase Reporters**

| **Gene** | **Sequences** |
| --- | --- |
| EIF4B-wt | Forward:5’-ATACGCGTTTTTCCACTCTTGGAAAGGTAG-3’  Reverse: 5’- ATGTTTAAACCTCCCAGGTAATAAGTCTAC-3’ |
| EIF4B-mut1 | Forward: 5’-TAACCCCCCAGGGGGGTAGTTATAATTGAGACTATAGGCCATAAAG-3’  Reverse:5’- CTTTATGGCCTATAGTCTCAATTATAACTACCCCCCTGGGGGGTTA-3’ |
| EIF4B-mut2 | Forward: 5’- GGGACAGGGACAGTTAAATTATAATCCTTTCTTACAACCTTGATG-3’  Reverse:5’- CATCAAGGTTGTAAGAAAGGATTATAATTTAACTGTCCCTGTCCC-3’ |
| EIF4B-mut3 | Forward: 5’- ATGTTTAAACATTATAGGTAATAAGTCTACAGATTAC-3’  Reverse:5’- GTAATCTGTAGACTTATTACCTATAATGTTTAAACAT-3’ |
| FOXO4-wt | Forward:5’- ATACGCGTCTGTTGGAAATGTGAAGTC-3’  Reverse:5’- ATGTTTAAACTTCCAGGCCTTCAATGTACT-3’ |
| FOXO4-mut1 | Forward:5’- GTGGCCTTACCCCTGCCTTTATAATCAGGATTTTTTTGTAGAG-3’  Reverse:5’- CTCTACAAAAAAATCCTGATTATAAAGGCAGGGGTAAGGCCAC-3’ |
| FOXO4-mut2 | Forward:5’-CATGGGTTAGTGCTATGGTATAATAGTACATTGAAGGCCTGG-3’  Reverse:5’- CCAGGCCTTCAATGTACTATTATACCATAGCACTAACCCATG-3’ |
| PRKCA-wt | Forward:5’- ATACGCGTAAGCCAGTGTGTACATATG-3’  Reverse:5’- ATGTTTAAACTACTCTGAGGACCTTGCATG-3’ |
| PRKCA-mut1 | Forward:5’- CATATGTTCATTTTAATCTCTATAATATTATTTTTCCATCCAGGGTG-3’  Reverse:5’- CACCCTGGATGGAAAAATAATATTATAGAGATTAAAATGAACATATG-3’ |
| PRKCA-mut2 | Forward:5’- GGCATTGGCCTTTCCAGTCTTATAATGAGCGCGCTGCTTTGGTGAG-3’  Reverse:5’- CTCACCAAAGCAGCGCGCTCATTATAAGACTGGAAAGGCCAATGCC-3’ |
| TET3-wt | Forward:5’- ATACGCGTCTGTCCTCTCTGATAGG-3’  Reverse:5’- ATGTTTAAACCTCCCACAATTACATGTTAAAACATAC-3’ |
| TET3-mut1 | Forward:5’- CTTCTGTCCTCTCTGATAGGATATAATAGTCTGCAGAAAACCATCTG-3’  Reverse:5’- CAGATGGTTTTCTGCAGACTATTATATCCTATCAGAGAGGACAGAAG-3’ |
| TET3-mut2 | Forward:5’- ATGTTTAAACATTATACAATTACATGTTAAAACATAC-3’  Reverse:5’- GTATGTTTTAACATGTAATTGTATAATGTTTAAACAT-3’ |
