# Supplementary material for: miR-150 exerts antileukemia activity in vitro and in vivo through regulating genes in multiple pathways
Source: Cell Death Dis. 2016 Sep 22;7(9):e2371–. doi: 10.1038/cddis.2016.256 (PMC5059860; doi:10.1038/cddis.2016.256)
Supplement: Supplementary Table 4 [file cddis2016256x9.doc]

**Table S4 shRNA sequences used for knockdown experiment**

| **Target Gene** | **Sequences** |
| --- | --- |
| *EIF4B* | 5’-gatccccCCAACTTCTAAACCTCCCAAAttcaagagaTTTGGGAGGTTTAGAAGTTGGtttttggaa -3’ |
| *FOXO4* | 5’-gatccccGCTGTTAGATGGGCTCAATCTttcaagagaAGATTGAGCCCATCTAACAGCtttttggaa -3’ |
| *PRKCA* | 5’-gatccccGCAAAGGACTGATGACCAAACttcaagagaGTTTGGTCATCAGTCCTTTGCtttttggaa -3’ |
| *TET3* | 5’-gatccccGCGATTGCGTCGAACAAATAGttcaagagaCTATTTGTTCGACGCAATCGCtttttggaa -3’ |
